# Predicting mortality with the international classification of disease injury severity score using survival risk ratios derived from an Indian trauma population: A cohort study

**DOI:** 10.1371/journal.pone.0199754

**Published:** 2018-06-27

**Authors:** Jonatan Attergrim, Mattias Sterner, Alice Claeson, Satish Dharap, Amit Gupta, Monty Khajanchi, Vineet Kumar, Martin Gerdin Wärnberg

**Affiliations:** 1 Department of Public Health Sciences, Karolinska Institutet, Stockholm, Sweden; 2 Department of General Surgery, Lokmanya Tilak Municipal Medical College & General Hospital, Mumbai, India; 3 Division of Trauma Surgery & Critical Care, J.P.N. Apex Trauma Center, New Delhi, India; 4 Department of General Surgery, Seth GS Medical College and KEM Hospital, Mumbai, India; University of Texas Health Science Center at Houston, UNITED STATES

## Abstract

**Background:**

Trauma is predicted to become the third leading cause of death in India by 2020, which indicate the need for urgent action. Trauma scores such as the international classification of diseases injury severity score (ICISS) have been used with great success in trauma research and in quality programmes to improve trauma care. To this date no valid trauma score has been developed for the Indian population.

**Study design:**

This retrospective cohort study used a dataset of 16047 trauma-patients from four public university hospitals in urban India, which was divided into derivation and validation subsets. All injuries in the dataset were assigned an international classification of disease (ICD) code. Survival Risk Ratios (SRRs), for mortality within 24 hours and 30 days were then calculated for each ICD-code and used to calculate the corresponding ICISS. Score performance was measured using discrimination by calculating the area under the receiver operating characteristics curve (AUROCC) and calibration by calculating the calibration slope and intercept to plot a calibration curve.

**Results:**

Predictions of 30-day mortality showed an AUROCC of 0.618, calibration slope of 0.269 and calibration intercept of 0.071. Estimates of 24-hour mortality consistently showed low AUROCCs and negative calibration slopes.

**Conclusions:**

We attempted to derive and validate a version of the ICISS using SRRs calculated from an Indian population. However, the developed ICISS-scores overestimate mortality and implementing these scores in clinical or policy contexts is not recommended. This study, as well as previous reports, suggest that other scoring systems might be better suited for India and other Low- and middle-income countries until more data are available.

## Introduction

Trauma was estimated to cause 4.8 million deaths in 2013, which is greater than the deaths caused by HIV/AIDS, tuberculosis, malaria and maternal conditions combined [1]. Ninety per cent of these deaths have been estimated to occur in low- and middle-income countries (LMICs) and an estimated 2 million lives could be saved annually by improving the quality of trauma care in LMICs [2,3].

India is an LMIC with more than 1 million annual trauma deaths, and by 2020, trauma is predicted to become the country’s third leading cause of death[1,4]. The majority of trauma victims are young and in their most productive phases of life, creating both physiological and psychological suffering as well as an economic drain [4–7]. Hence, efforts to strengthen trauma care and prevention in India are urgently needed [8].

Trauma patients constitute a heterogeneous population, making trauma research and outcome comparisons over time and between contexts challenging [9]. One crucial factor to consider in such comparisons is injury severity, and several scores have been developed for this purpose [7,10–13]. The use of these scores as part of quality improvement programmes has been associated with improved trauma care[14], but no optimal score exists for the Indian population [15,16].

The international classification of disease (ICD) injury severity score (ICISS) is a tool used to determine injury severity [12,17]. This score uses survival risk ratios (SRRs), empirically derived for each unique ICD code, to estimate a patient’s probability of survival. According to a recent systematic review, the ICISS outperforms many other scores [18], but little research on the ICISS is from LMICs. Since the ICISS is derived from a specific context [12], the current study sought to identify whether an Indian version of the ICISS could predict mortality in four public hospitals in urban India.

## Methods

### Study design

We analysed data from a previously conducted cohort study to derive and temporally validate a new version of the ICISS. This study was registered with clinicaltrials.gov with the registration number NCT02716649.

### Setting

We used data from the Towards Improved Trauma Care Outcomes in India (TITCO) project that were collected at four public university hospitals across urban India. The data used in this study were collected from the four study centres between July 2013 and December 2015. The four centres included Lokmanya Tilak Municipal General Hospital in Mumbai, King Edward Memorial Hospital in Mumbai, Jai Prakash Narayan Apex Trauma Center in Delhi, and the Institute of Post-Graduate Medical Education and Research and Seth Sukhlal Karnani Memorial Hospital in Kolkata.

At each hospital, one trained project officer with a health master’s degree or higher level of education collected all the data. The project officers worked eight hours a day rotating between day, evening and night shifts, and were continuously supervised and trained by project management. Patients were followed up until discharge, death or 30 days.

### Participants and eligibility criteria

Patients with a history of trauma who arrived alive to the study hospitals and were admitted or died between arrival and admission were included. Patients with isolated limb fractures without vascular injury were excluded because they are placed in the “orthopaedic pathway” instead of the “trauma pathway” in the study hospitals, which made patient follow-up was impossible for these individuals.

The project officers included all consecutive patients who fit the eligibility criteria. Data for the patients admitted during the project officers’ shifts were collected using direct observation and extraction from patient records. Data for patients admitted outside of their shifts were collected retrospectively from patient records within days of patient arrival.

### Outcomes variables and covariates

The primary and secondary outcomes were in hospital death at any of the hospitals participating in this study within 30 days and 24 hours respectively. The explanatory variable was the ICISS. Other covariates included the date and time of arrival, age, sex, mechanism of injury and transfer status. All quantitative variables were analysed as continuous. We used these variables to characterize the study sample.

### Data sources/measurements

Free-text injury descriptions were extracted from patient records, including imaging reports and surgical notes, and were then coded using the ICD-10. We calculated the SRR for each unique ICD-10 code using $SRR=\frac{A}{A+B}$, where A denotes the number of surviving patients and B denotes the number of non-surviving patients with the same specific ICD-code. The calculated SRR wasassigned a value between 0 and 1, where 1 represents 100% survival and 0 represents 0% survival. The final ICISS score for each patient was calculated as the product of all individual SRRs. Hence, the ICISS also ranges from 0 to 1 and should be interpreted as the patient’s probability of survival. This method is commonly referred to as the conventional or multiplicative ICISS.

### Bias

During the conversion from free-text injury descriptions to ICD codes, the coders were blinded to patient demographics and outcomes. ICD coding was conducted after the coders completed the World Health Organization (WHO) ICD-10 online training module and achieved more than 80% agreement with an external experienced coder over several samples of 50 injuries.

### Study size

We used all available data from TITCO and created a temporally split sample, henceforth referred to as the derivation sample and validation sample, using the earlier data for derivation and the most recent data for validation. The validation sample size was estimated by including the most recent 200 consecutive events, i.e. patients who died within 24 hours, and all non-events enrolled during the same time period [19,20]. We used mortality within 24 hours for our sample size calculation because we wanted the study to be powered for secondary outcomes as well. This effective sample size allowed us to detect a significant difference in the discrimination and calibration of the ICISS between the derivation and calibration samples at 80% power and a 5% significance level. All remaining patients constituted the derivation sample.

### Statistical methods and analyses

The derivation and validation of the ICISS were conducted as two separate steps using R for all statistical analyses [21]. We assessed predictive performance in terms of discrimination, by calculating the area under the receiver operating characteristics curve (AUROCC) and calibration by visually comparing the observed and predicted outcomes in a calibration plot and calculating the calibration slope and intercept. Confidence intervals for predictive performance measures were estimated using a bootstrap approach [22].

We interpreted overlapping 95% confidence intervals (CI) as evidence of lack of a statistically significant difference. Parametric and non-parametric exact tests were used as appropriate, with a 5% significance level. Our main analysis was a complete case analysis, in which we excluded observations with missing values in any of the outcomes or covariates. Observations with no injuries reported were assigned an ICISS of 1, and for each observation, the final ICISS was calculated based only on the SRRs for ICD codes that occurred in at least 10 observations in the derivation sample. Ten was chosen as a compromise between precision and the available ICD-codes.

### Derivation and validation

We derived SRRs in the derivation sample for each of the outcomes, mortality within 30 days and within 24 hours, and used these ratios to calculate two ICISSs for each patient. The SRRs for 30-day mortality are referred to as SRRm30d, and the SRRs for 24-hour mortality are referred to as SRRm24h. We used similar notation to refer to the ICISS, i.e., ICISSm30d and ICISSm24h. Finally, we assessed the performance of ICISSm30d in predicting mortality within 30 days and within 24 hours and repeated this analysis for ICISSm24h. We used the SRRm30d and SRRm24h results from the derivation sample to calculate ICISSm30d and ICISSm24h in the validation sample. Performance was assessed in the same manner as in the derivation sample, and the results were compared to those from the derivation sample.

### Sensitivity analyses

We conducted four sensitivity analyses. The first analysis included observations with missing values in covariates but with complete outcome data. The second excluded observations without any reported injury, which were previously assigned an ICISS value of 1 regardless of outcome. The third calculated ICISS was based on all available calculated SRRs, regardless of how frequently the corresponding ICD codes occurred in the dataset. Finally, we calculated the ICISS for each patient based only on unique ICD codes, in other words, each ICD code was allowed to contribute only one SRR to the ICISS even if it occurred more than once in the same patient.

### Ethical considerations

Ethics committees at all participating centres approved the collation of the database and granted a waiver of consent for the trauma patients. This study was conducted using anonymized data. The names of the review boards and ethical approval reference numbers for the original study that collected the data were as follows: Seth GS Medical College and King Edward Memorial Hospital Institutional Ethics Committee, approval number IEC(I)/OUT/222/14; Lokmanya Tilak Municipal Medical College & Lokmanya Tilak Municipal General Hospital Institutional Ethics Committee, approval number IEC/11/13; Institute of Post-Graduate Medical Education and Research Institutional Ethics Committee, approval number IEC/279; and All India Institute of Medical Sciences Institutional Ethics Committee, approval number IEC/NP-279/2013 RP-Ol/2013.

## Results

### Participants

Between July 2013 and December 2015, a total of 16047 patients were included in the TITCO dataset. The complete case analysis excluded patients whose records had missing values in covariates or outcomes, leaving 15,865 patients for the analysis.

### Descriptive data

The complete case sample (n = 15,865) was split into a derivation sample (n = 11,944) and a validation sample (n = 3,921). Age and gender showed the lowest numbers of missing values. Date of arrival to the hospital and mechanism of injury showed the highest number of missing values with 95 (0.6%) and 60 (0.4%) missing values, respectively (Table 1).

**Table 1:** Missing data per variable.

Variables	Number of missing values (%)
Age	0 (0)
Gender	0 (0)
Transfer status	36 (0.22)
Mechanism of injury	60 (0.37)
30 day mortality	21 (0.13)
24 hour mortality	21 (0.13)
Date of arrival	95 (0.6)

The mean age was 31.6 (SD 18.9, range 0–97) years in the derivation sample and 33.3 (SD 18.7, range 0–95) years in the validation sample. The mean age for non-survivors was higher than that for survivors, at 37 (SD 18.9, range 1–97) and 38 years, respectively. The majority of patients were male (73.8% and 79.3% in the derivation and validation samples, respectively). Road traffic injury (41% and 46.9%) was the most common cause of trauma followed by falls (33% and 31.2%). The majority (70.4% and 73.3%) of patients were transferred from another hospital (Table 2).

**Table 2:** Patient characteristics.

	Derivation sample			Validation sample		
Variable	Patients who survived(n = 9269)	Patients who died (n = 2675)	All patients (n = 11944)	Patients who survived(n = 3137)	Patients who died (n = 784)	All patients (n = 3921)
Mean age (SD)	30 (18.6)	37 (19.2)	31.6 (18.9)	32.1 (18.3)	38 (19.7)	33.3 (18.7)
**Gender**						
Female (%)	2065 (22.3)	702 (26.2)	2767 (23.2)	649 (20.7)	649 (20.7)	833 (21.2)
Male (%)	7204 (77.7)	1973 (73.8)	9177 (76.8)	2488 (79.3)	2488 (79.3)	3088 (78.8)
**Mechanism of Injury**						
Assault (%)	792 (8.5)	89 (3.3)	881 (7.4)	247 (7.9)	19 (2.4)	266 (6.8)
Burn (%)	560 (6)	438 (16.4)	998 (8.4)	120 (3.8)	118 (15.1)	238 (6.1)
Fall (%)	3213 (34.7)	732 (27.4)	3945 (33)	1044 (33.3)	180 (23)	1224 (31.2)
Other (%)	439 (4.7)	52 (1.9)	491 (4.1)	155 (4.9)	25 (3.2)	180 (4.6)
Railway injury(%)	468 (5)	264 (9.9)	732 (6.1)	114 (3.6)	62 (7.9)	176 (4.5)
RTI*s (%)	3797 (41)	1100 (41.1)	4897 (41)	1457 (46.4)	380 (48.5)	1837 (46.9)
**Transfer status**						
No (%)	2862 (30.9)	673 (25.2)	3535 (29.6)	864 (27.5)	864 (27.5)	1048 (26.7)
Yes (%)	6407 (69.1)	2002 (74.8)	8409 (70.4)	2273 (72.5)	2273 (72.5)	2873 (73.3)

*RTIs = Road traffic injuries

### Outcome data

Out of the 15,865 patients in the complete case analysis, 3,459 (21.6%) patients died within 30 days and 1,025 (6.4%) died within 24 hours. Mortality was higher in the derivation sample (22.3%, n = 2675 for 30-day mortality; 6.9%, n = 825 for 24-hour mortality) than in the validation sample (20.0%, n = 784 for 30-day mortality and 5.1%, n = 200 for 24-hour mortality).

### Main results

The different combinations of ICISSm24h and ICISSm30d with m30d and m24h were assessed using calibration and discrimination. For each measure, a 95% confidence interval was generated using a bootstrap approach with 1000 draws with replacement of the same size as the original sample (Table 3).

**Table 3:** Discrimination and calibration for the complete case analysis.

Mortality time + ICISS score	Derivation sample			Validation sample		
	AUROCC	Calibration slope	Calibration intercept	AUROCC	Calibration slope	Calibration intercept
m30d + ICISSm30d	0.633 (0.620–0.646)	0.300 (0.271–0.333)	0.064 (0.047–0.08)	0.618 (0.597–0.638)	0.269 (0.219–0.325)	0.071 (0.046–0.096)
m30d + ICISSm24h	0.606 (0.594–0.619)	0.571 (0.511–0.634)	0.125 (0.113–0.136)	0.575 (0.552–0.596)	0.432 (0.313–0.545)	0.136 (0.117–0.157)
m24h + ICISSm24h	0.519 (0.496–0.541)	0.079 (0.041–0.12)	0.055 (0.047–0.063)	0.527 (0.48–0.569)	-0.007 (-0.073–0.059)	0.051 (0.039–0.062)
m24h + ICISSm30d	0.494 (0.460–0.510)	-0.01 (-0.03–0.01)	0.074 (0.062–0.086)	0.537 (0.49–0.608)	-0.022 (-0.054–0.011)	0.061 (0.042–0.077)

ICISS: International Classification of Disease Injury Severity Score, AUROCC: Area under the receiver operating characteristics curve, m30d: Mortality within 30 days, m24h: Mortality within 24 hours

**Fig 1:**
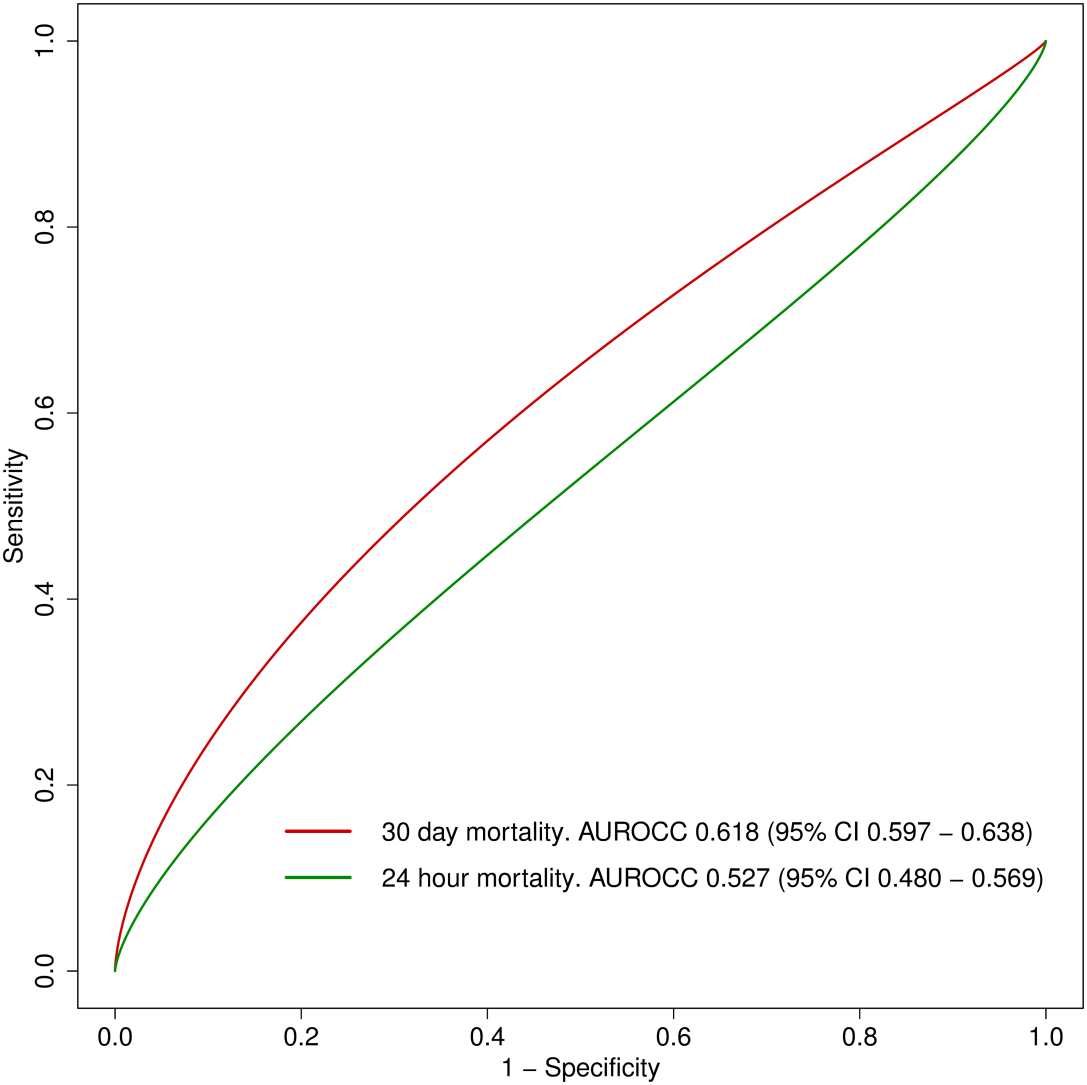
Area under the receiver operating characteristics curve (AUROCC) for ICISSm30d and ICISSm24h.

The AUROCC of ICISSm30d in predicting 30-day mortality in the derivation sample was 0.633 (95% CI 0.620–0.646), and the calibration slope was 0.300 (95% CI 0.271–0.333), suggesting that ICISSm30d substantially overestimated the risk of mortality (Fig 1). The calibration slope in the derivation sample for ICISSm24h on 24-hour mortality was 0.079 (95% CI 0.041–0.12) with an AUROCC of 0.519 (95% CI 0.496–0.541), indicating a prediction value similar to chance alone. The results from the derivation sample were largely mimicked in the validation sample.

**Fig 2:**
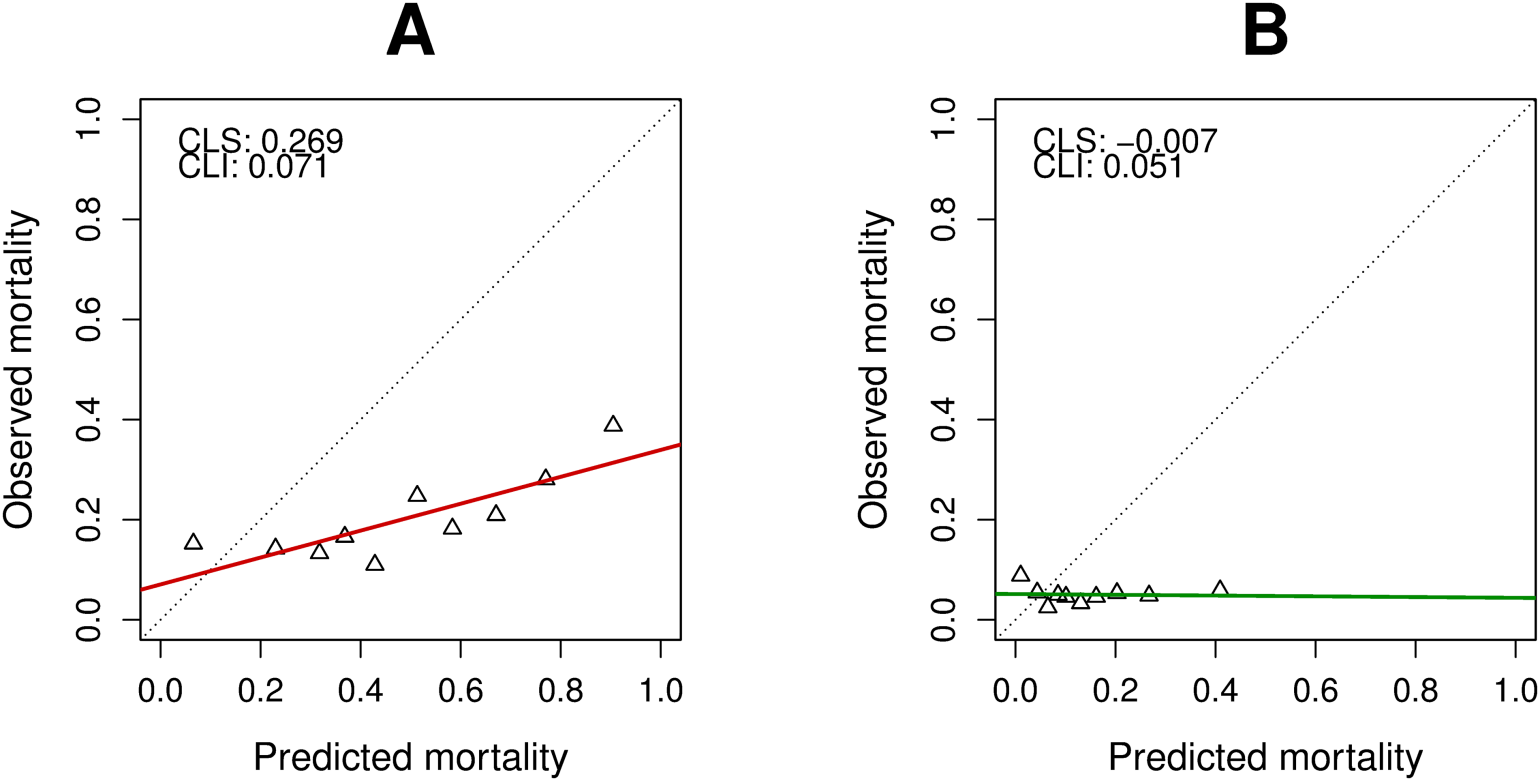
Calibration plots comparing observed and predicted mortality. Plots for the validation sample are shown for **(A)** within 30 days and **(B)** within 24 hours. Abbreviations: CSL: Calibration line slope, CLI: Calibration line intercept.

Calibration plots were created in which the ICISS scores were split into deciles and plotted against mortality (Fig 2). The main analysis was conducted in the validation sample for ICISSm30d with m30d and ICISSm24h with m24h. Fig 2A shows a linear association between ICISSm30d and mortality, and the point estimates are relatively close to the solid line. The calibration slopes capture the overall trend but with substantial overestimation of mortality. The point estimates in Fig 2B are linear, but the calibration slope suggests no or even a slight inverse relationship.

### Sensitivity analyses

The results from the sensitivity analysis did not differ significantly from the main analysis, for more information see the supplementary material (S1–S4 Tables).

## Discussion

### Key results

Our aim was to derive and validate two versions of the ICISS for 30-day mortality (ICISSm30d) and 24-hour mortality (ICISSm24h) using SRRs derived from trauma patients admitted to four public university hospitals in urban India. Our results indicate that neither of the developed scores performs adequately in terms of discrimination and calibration when used to predict death at eather 30 days or 24 hours. To illustrate this point, the best discriminating score in the validation sample was for ICISSm30d when used to predict death at 30 days, and this score achieved an AUROCC of only 0.618, with accompanying poor estimates of calibration.

### Limitations

This study was based on the Largest Indian trauma dataset available, but the sample size of 16,000 patients is still small compared with similar studies from other contexts in which more than 100,000 patients were used [23,24]. The comparably small sample size may have resulted in more unstable SRRs. To address this limitation, we used SRRs from injuries that occurred 10 or more times, but this cut-off should be increased in future studies when more data are available.

The clinical environment studied comes with limitations including a lack of diagnostic tools, which is likely to result in undocumented injuries[16]. As previous literature suggests, this limitation may result in lower SRRs for minor injuries due to the presence of more severe, co-existing undocumented injuries, which would in turn translate into an overestimation of mortality, especially for ICISSm24h where missed diagnoses are common [23].

We studied only the conventional ICISS in which all of a patient’s injuries are accounted for in the final score. The study by Kilgo PD et al. evaluated methods using a maximum amount of injuries or just the worst injury [24], and the methods using just the worst injury outperformed models using multiple or all injuries, suggesting that the model in this study, the conventional ICISS, was not the optimal model [24,25].

### Interpretation

Our study demonstrates the challenges associated with deriving local SRRs using a relatively limited sample. It is notable however, that this dataset is to our knowledge the largest available trauma dataset from India, the world’s second most populous country. It is unlikely that much larger datasets exist from many other LMICs; thus deriving local SRRs in similar contexts may be equally challenging.

Recent research evaluated the performance of five different trauma scores in part of our dataset [16]. The scores evaluated were the injury severity score (ISS), the new injury severity score (NISS), the revised trauma score (RTS), the Kampala trauma score (KTS), and the trauma injury severity score (TRISS). Like the ICISS, the ISS and NISS are purely anatomical scores, whereas the RTS is a physiological score, and the KTS and TRISS are combined scores, i.e., including both anatomical and physiological components. In that study, TRISS discriminated best with an AUROCC of 0.82 and was closely followed by RTS at 0.81. The former score is a composite of the RTS, ISS, age and blunt or penetrating injury, indicating that the addition of ISS, age and injury type results in a negligible improvement in discrimination. These results suggest that physiological scores such as the RTS would be preferable in LMICs, provided issues with vital sign registration frequency are corrected.

Further, research on an earlier dataset from Mumbai showed that physiological scores outperformed anatomical scores [15]. The ISS was the worst discriminating score, with an AUROCC of 0.69, i.e., similar to that of the ICISS in our study. It is likely that undocumented injuries and incomplete imaging contributed to the poor performance of the ISS in that study and in our study. Interestingly however, even in more complete datasets, such as that of San Francisco General Hospital, the discrimination of physiological scores approaches that of combined scores [26], indicating that the former may be a more stable choice.

### Generalizability

Transfer of ICISS scores between countries and contexts has been attempted in high-income countries with mixed results [27]. Pooling data from different countries to create a multinational ICISS also reduced performance [28]. Thus, the SRRs derived in our study are unlikely to transfer well to other settings, even if they had displayed better performance in our data. Instead, our research highlights the importance of validating an ICISS generated using locally derived SRRs in an independent sample before applying it to practice. As we show, using the SRRs derived here would result in a serious overestimation of mortality.

### Conclusion

The scores developed in this study systematically overestimated 30-day mortality, and prediction of 24-hour mortality suggests that an ICISS based on the SRRs derived in this study should not be used to predict short-term mortality at all. Due to poor discrimination and calibration, the current models cannot be considered a reliable choice of trauma score in the studied context. Our study highlights the challenges associated with deriving local SRRs based on limited data, and the findings suggest that other trauma scores should be considered in settings where only small samples are available to calculate SRRs. Other LMICs face similar challenges as India, suggesting that resources and research might be better directed towards other scoring systems than the ICISS.

## Supporting information

S1 Table Sensitivity analysis I. Including patients with missing covariates. ICISS: International classification of disease injury severity score, AUROCC: Area under the receiver operating characteristic curve, m30d: Mortality within 30 days, m24h: Mortality within 24 hours.
(DOC)

S2 Table Sensitivity analysis II. Excluding patients without observed injuries. ICISS: International classification of disease injury severity score, AUROCC: Area under the receiver operating characteristic curve, m30d: Mortality within 30 days, m24h: Mortality within 24 hours.
(DOC)

S3 Table Sensitivity analysis III. The ICISS was based on all SRRs independent of how many patients they were based on. ICISS: International classification of disease injury severity score, AUROCC: Area under the receiver operating characteristic curve, m30d: Mortality within 30 days, m24h: Mortality within 24 hours.
(DOC)

S4 Table Sensitivity analysis IV. A given ICD code could contribute only once to the patient’s final ICISS score. ICISS: International classification of disease injury severity score, AUROCC: Area under the receiver operating characteristic curve, m30d: Mortality within 30 days, m24h: Mortality within 24 hours.
(DOC)

## Abbreviations

AUROCC: Area under the receiver operating characteristics curve
CI: Confidence interval
CLI: Calibration line intercept
CSL: Calibration line slope
ICD: International Classification of Diseases
ICISS: International Classification of Disease Injury Severity Score
LMICS: low- and middle-income
SD: Standard deviation
SRR: Survival risk ratios
TITCO: Towards Improved Trauma Care Outcomes
