# Supplementary material for: Predicting mortality with the international classification of disease injury severity score using survival risk ratios derived from an Indian trauma population: A cohort study
Source: PLoS One. 2018 Jun 27;13(6):e0199754. doi: 10.1371/journal.pone.0199754 (PMC6021077; doi:10.1371/journal.pone.0199754)
Supplement: S1 Table — Including patients with missing covariates. ICISS: International classification of disease injury severity score, AUROCC: Area under the receiver operating characteristic curve, m30d: Mortality within 30 days, m24h: Mortality within 24 hours. (DOC) [file pone.0199754.s001.doc]

|  | | | | | | |
| --- | --- | --- | --- | --- | --- | --- |
| **S_table.1 : Discrimination and Calibration for sensitivity analysis** I | | | | | | |
| **Mortality time + ICISS score** | **Derivation sample** | | | **Validation sample** | | |
|  | AUROCC | Calibration Slope | Calibration intercept | AUROCC | Calibration Slope | Calibration intercept |
| m30d + ICISSm30d | 0.634 (0.621-0.646) | 0.301 (0.27-0.332) | 0.064 (0.048-0.08) | 0.619 (0.595-0.641) | 0.271 (0.218-0.328) | 0.07 (0.044-0.097) |
| m30d + ICISSm24h | 0.606 (0.594-0.619) | 0.569 (0.508-0.631) | 0.126 (0.114-0.138) | 0.577 (0.553-0.6) | 0.437 (0.311-0.55) | 0.136 (0.118-0.156) |
| m24h + ICISSm24h | 0.518 (0.494-0.54) | 0.079 (0.04-0.122) | 0.055 (0.046-0.063) | 0.527 (0.479-0.569) | -0.009 (-0.075-0.06) | 0.052 (0.04-0.063) |

*_*
