# Supplementary material for: Predicting mortality with the international classification of disease injury severity score using survival risk ratios derived from an Indian trauma population: A cohort study
Source: PLoS One. 2018 Jun 27;13(6):e0199754. doi: 10.1371/journal.pone.0199754 (PMC6021077; doi:10.1371/journal.pone.0199754)
Supplement: S2 Table — Excluding patients without observed injuries. ICISS: International classification of disease injury severity score, AUROCC: Area under the receiver operating characteristic curve, m30d: Mortality within 30 days, m24h: Mortality within 24 hours. (DOC) [file pone.0199754.s002.doc]

|  | | | | | | |
| --- | --- | --- | --- | --- | --- | --- |
| **S_table.2 : Discrimination and Calibration for sensitivity analysis** II***** | | | | | | |
| **Mortality time + ICISS score** | **Derivation sample** | | | **Validation sample** | | |
|  | AUROCC | Calibration Slope | Calibration intercept | AUROCC | Calibration Slope | Calibration intercept |
| m30d + ICISSm30d | 0.664 (0.653-0.677) | 0.4 (0.368-0.43) | -0.004 (-0.02-0.015) | 0.647 (0.625-0.67) | 0.356 (0.305-0.417) | 0.016 (-0.014-0.042) |
| m30d + ICISSm24h | 0.634 (0.622-0.647) | 0.679 (0.617-0.741) | 0.095 (0.082-0.108) | 0.598 (0.575-0.62) | 0.515 (0.382-0.635) | 0.117 (0.098-0.14) |
| m24h + ICISSm24h | 0.61 (0.59-0.632) | 0.177 (0.138-0.216) | 0.028 (0.021-0.035) | 0.562 (0.519-0.649) | 0.071 (0.004-0.131) | 0.032 (0.021-0.042) |
| m24h + ICISSm30d | 0.581 (0.561-0.603) | 0.063 (0.045-0.081) | 0.024 (0.014-0.034) | 0.549 (0.504-0.598) | 0.034 (0.006-0.067) | 0.026 (0.008-0.04) |
