# Supplementary material for: Predicting mortality with the international classification of disease injury severity score using survival risk ratios derived from an Indian trauma population: A cohort study
Source: PLoS One. 2018 Jun 27;13(6):e0199754. doi: 10.1371/journal.pone.0199754 (PMC6021077; doi:10.1371/journal.pone.0199754)
Supplement: S3 Table — The ICISS was based on all SRRs independent of how many patients they were based on. ICISS: International classification of disease injury severity score, AUROCC: Area under the receiver operating characteristic curve, m30d: Mortality within 30 days, m24h: Mortality within 24 hours. (DOC) [file pone.0199754.s003.doc]

|  | | | | | | |
| --- | --- | --- | --- | --- | --- | --- |
| **Table 6: Discrimination and Calibration for sensitivity analysis** III ***** | | | | | | |
| **Mortality time + ICISS score** | **Derivation sample** | | | **Validation sample** | | |
|  | AUROCC | Calibration Slope | Calibration intercept | AUROCC | Calibration Slope | Calibration intercept |
| m30d + ICISSm30d | 0.638 (0.625-0.651) | 0.312 (0.276-0.34) | 0.056 (0.041-0.076) | 0.619 (0.596-0.642) | 0.269 (0.221-0.324) | 0.069 (0.04-0.094) |
| m30d + ICISSm24h | 0.608 (0.595-0.621) | 0.571 (0.509-0.631) | 0.123 (0.112-0.135) | 0.574 (0.552-0.598) | 0.41 (0.301-0.519) | 0.138 (0.117-0.158) |
| m24h + ICISSm24h | 0.53 (0.507-0.555) | (0.057-0.141) | 0.051 (0.043-0.06) | 0.526 (0.481-0.568) | -0.007 (-0.068-0.055) | 0.051 (0.038-0.062) |
| - 1. m24h + ICISSm30d | 0.502 (0.476-0.521) | -0.005 (-0.028-0.015) | 0.072 (0.06-0.086) | 0.530 (0.483-0.593) | 0.019 (-0.05-0.014) | 0.06 (0.042-0.077) |
