# Supplementary material for: Predicting mortality with the international classification of disease injury severity score using survival risk ratios derived from an Indian trauma population: A cohort study
Source: PLoS One. 2018 Jun 27;13(6):e0199754. doi: 10.1371/journal.pone.0199754 (PMC6021077; doi:10.1371/journal.pone.0199754)
Supplement: S4 Table — A given ICD code could contribute only once to the patient’s final ICISS score. ICISS: International classification of disease injury severity score, AUROCC: Area under the receiver operating characteristic curve, m30d: Mortality within 30 days, m24h: Mortality within 24 hours. (DOC) [file pone.0199754.s004.doc]

|  | | | | | | |
| --- | --- | --- | --- | --- | --- | --- |
| **Table 7: Discrimination and Calibration for sensitivity analysis** IV***** | | | | | | |
| **Mortality time + ICISS score** | **Derivation sample** | | | **Validation sample** | | |
|  | AUROCC | Calibration Slope | Calibration intercept | AUROCC | Calibration Slope | Calibration intercept |
| m30d + ICISSm30d | 0.633 (0.621-0.645) | 0.31 (0.281-0.341) | 0.065 (0.048-0.081) | 0.619 (0.596-0.642) | 0.273 (0.207-0.323) | 0.074 (0.05-0.11) |
| m30d + ICISSm24h | 0.605 (0.593-0.617) | 0.622 (0.559-0.686) | 0.123 (0.11-0.135) | 0.574 (0.55-0.597) | 0.465 (0.331-0.593) | 0.134 (0.114-0.155) |
| m24h + ICISSm24h | 0.52 (0.496-0.542) | 0.095 (0.05-0.139) | 0.053 (0.045-0.061) | 0.527 (0.481-0.567) | 0.003 (-0.067-0.076) | 0.049 (0.036-0.06) |
| m24h + ICISSm30d | 0.495 (0.46-0.505) | -0.008 (-0.029-0.012) | 0.073 (0.061-0.084) | 0.536 (0.489-0.594) | -0.026 (-0.061-0.006) | 0.063 (0.046-0.083) |
